# Married Adults Coresiding with Older Parents: Implications for Paid Work and Domestic Workloads

**DOI:** 10.1007/s12062-021-09346-2

**Published:** 2021-08-20

**Authors:** Ekaterina Hertog, Man-Yee Kan

**Affiliations:** University of Oxford, Oxford, UK

## Abstract

The rise of life expectancy throughout the developed world has meant that older adults play an increasingly important role in their grown-up children’s lives. We evaluate whether the intergenerational solidarity theory is useful for understanding the intergenerational transfers of time in Japan given the relatively generous welfare provision for the older adults and the fall in intergenerational coresidence. We apply seemingly unrelated regression models to data of the 2006 Japanese Survey on Time Use and Leisure Activities (Statistics Bureau Japan, 2006) to investigate how coresidence patterns are associated with paid and unpaid work time of adult married children. The sample contains 23,226 married couples where both husband and wife are aged 20 to 59. We find evidence of intergenerational solidarity in coresident households. We also find that “doing gender” is layered through intergenerational exchanges of support between married working-age children and their older parents. Working-age women’s time use patterns are associated with coresidence arrangements and care needs of their older relatives to a much greater extent than working-age men’s. The observed patterns are consistent with healthy older women supporting their daughters’ careers in exchange for care when they need help themselves. For working-age men, the patterns are not very pronounced. Notably, working-age husbands without children appear to be more responsive to their older the parents’ care needs, suggesting that fatherhood may be associated with solidifying gendered role performance within Japanese couples.

## Introduction

Coresidence with older parents has attracted the attention of many researchers in recent decades. Increasing life expectancy together with the falling birth rate have resulted in a rising number of older adults across the developed world (Population Division, 2020). The unprecedented increase in the number of men and women aged 65 or above happened at the same time as several dramatic shifts in family behaviours occur. These include falling family sizes, a rise in women’s educational attainment and labour market participation (Fingerman et al., 2020). In this context, older adults can play two roles in their extended families. They can support their adult children as well as these children’s own families. Such help can make a big difference for adult children’s ability to combine work and family, especially when they start having children of their own. At the same time, as the older adults get older and frailer, they require care themselves. The latter scenario has given rise to widespread concerns about the sandwich generation in which adult children face double care responsibilities as they need to care for young children and frail older adults at the same time, potentially undermining their ability to succeed in the labour market (e.g., Vlachantoni et al., 2020). At least one recent paper hypothesises that coresidence or proximate residence with older parents in poor health may contribute to family resource dilution and is potentially linked to worse outcomes for grandchildren (Wang & Raymo, 2020). Generous state welfare provision for dependents can also weaken the associations between coresidence arrangements paid and unpaid work participation.

This paper aims to provide a comprehensive account of variation in married men’s and women’s paid work, housework and care work time using large scale national survey data from Japan. In the early twenty-first century Japan is characterised by a dramatic expansion of older-adult care coupled with relatively low spending on childcare (Estévez-Abe & Kim, 2014; Estévez-Abe & Naldini, 2016), potentially resulting in an imbalance of care needs between working-age adults and their parents.

We investigate how coresidence patterns and older adults’ care needs are associated with working-age couples’ paid work time as well as time spent on housework and childcare, and older-adult care by married men and women. Previous studies on the gender division of labour in a marriage focused predominantly on the dyadic interactions between husbands and wives. This paper addresses a research gap by emphasising the roles of older parents.

The paper is divided into three sections. First is the literature review, which includes (a) a summary of theoretical approaches that can help us make sense of the associations between coresidence patterns and care responsibilities on working-age adults’ paid and unpaid work participation; (b) a discussion of existing empirical findings and gaps in the literature and (c) a description of the Japanese context. This is followed by the data and methods section. The paper concludes with a discussion of our results and a consideration of the role coresidence continues to play in Japanese families.

## Background and Literature Review

In European and Anglophone societies, coresidence of adult children with older parents is not very common, though several countries have documented upward trends in recent years. In the US 18% of all households include at least two adult generations. In EU countries extended family household prevalence ranges from 0.1% of all households in the Netherlands and Denmark to 20% of all households in Bulgaria. Former Eastern European countries and to a lesser extent Southern European countries are characterised by particularly high rates of multigenerational coresidence in Europe (Iacovou & Skew, 2011; Kalmijn & Saraceno, 2008).

East Asian countries are generally characterised by higher levels of intergenerational coresidence compared to European and Anglophone societies. According to a recent UN report, 33% of those aged 60 or older lived independently (alone or with a spouse) in China in 2000; 35% in Hong Kong in 2011; 49% in South Korea in 2005; and 51% in Japan in 2010. The rest resided with their children. By comparison, in 2011 in Finland and France 87% of those aged 60 or over lived alone or with a spouse; in Germany, the figure was 90%, in Italy 70%, in Spain 58%, in Norway and the UK 86%, in the US 71% and Canada 74% (Department of Economic & Social Affairs, 2017). Although the prevalence of coresidence has been falling in Japan over time, it is still a common practice even among the young older adults aged 65–74. Importantly, this decline in coresidence has been accompanied by a steady increase in the prevalence of residential proximity to parents among recently married men and women in Japan (Kato, 2013; Wang & Raymo, 2020). Several generations of one family residing nearby are likely to support each other with unpaid work in ways somewhat similar to residing households (e.g. Chen et al, 2000; Raymo et al., 2010).

How people spend time is influenced by their household compositions. Larger households may have greater potential for economies of scale when it comes to unpaid work. Some members of the household may specialise in unpaid work to reduce the domestic work burden for the rest of the family, especially once new families are formed by adult children. Transitions into marriage (or a stable partnership) and parenthood are typically associated with dramatic changes in domestic workload and a rise in conflict between paid and unpaid work (Borra et al., 2020; Kan, 2009; Zhou & Kan, 2019). Moreover, sometimes several generations may choose to live together because a family member needs care (Takeda et al., 2004). In this scenario, multigenerational living may be associated with an increased need for unpaid work by other household members.

**Fig. 1:**
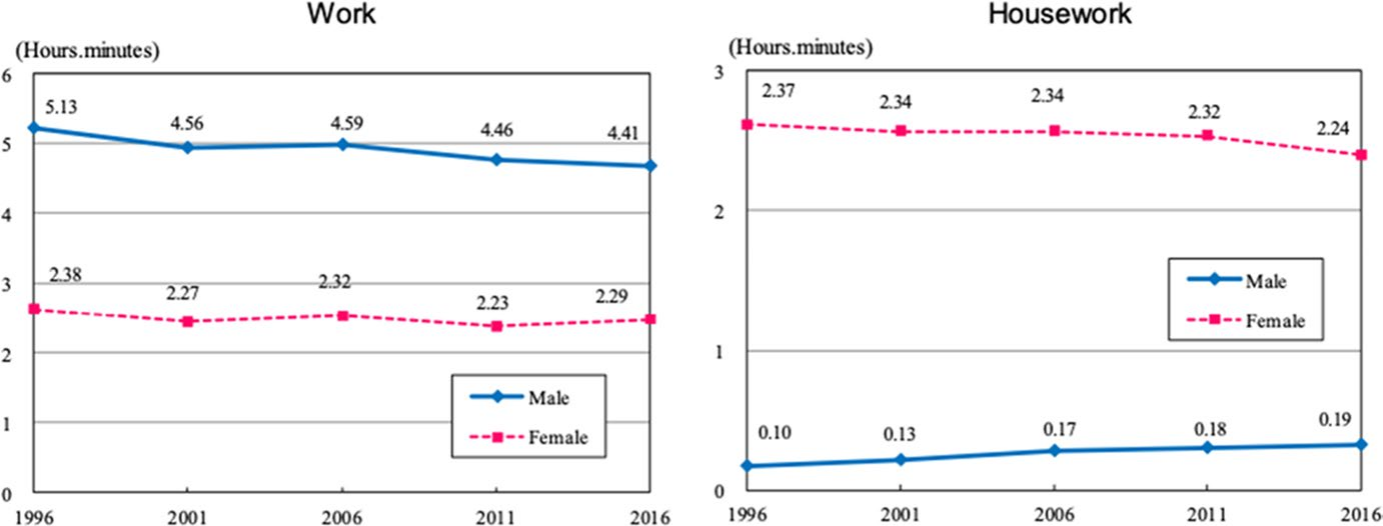
Time spent on paid work and housework by gender (1996–2016). Source: Copied directly from “2016 Survey on Time Use and Leisure Activities: Summary of results” https://www.stat.go.jp/english/data/shakai/2016/pdf/timeuse-a2016.pdf

While household composition influences the amount of required unpaid work, gender plays a major role in the way this work is shared. Marriage and children are always associated with the rise in time spent on housework for women (see Sayer, 2010 for a summary of evidence from Western countries; for evidence from East Asia see Sechiyama, 2013). There is a consistent negative association between motherhood and women’s employment (Ahn & Mira, 2002; Miller, 2011), testifying to the rise in intensity in the work-family conflict for women once they have children. For men, marriage and parenthood have historically been associated with decreases in time spent on housework, but this has changed in recent years. In many countries today, marriage is associated with increases in housework time for both women and men, although the change for women is larger while the effects for men tend to be modest. Having children leads to a rise in time spent on care work for both men and women (for a more detailed summary and references see Sayer, 2010). The division of labour in multigenerational households, therefore, is of particular interest in situations where there are children, and we compare families with and without underage children. This paper will focus on families of married adult children only, as these are individuals who have completed the transition to adulthood having formed their own families. We believe the dynamics associated with coresidence for these children are very different from those of unmarried adult children, who need to be analysed separately Fig. 1.

This study contributes a fuller understanding of variation in three major domains of time use (paid work, housework and childcare, and older-adult care) by the types of coresidence arrangements.

### Domestic Division of Labour Theories and Intergenerational Support in Multigenerational Households

Our conceptual approach will examine predictions about households where married adult children reside with their older parents from the *intergenerational solidarity perspective* and *the gender perspective*.

The *intergenerational solidarity theory* is specifically designed to make sense of intergenerational relations. In this framework, coresidence with one’s older parents or in-laws results in solidarity between older parents and their adult children(-in-law). This paper focuses on the instrumental aspect of the theory, which refers to non-financial mutual help between generations (e.g., cooking, cleaning, fetching groceries, providing care). According to this theory, we would expect that in households with more than one generation of adults, they will share unpaid work similarly to the ways they share financial and other resources (Shirahase & Raymo, 2014; Takagi & Silverstein, 2011). This approach predicts that the older generation will support their adult children as long as their health allows this. Once the older adults need help themselves, intergenerational solidarity will encourage their working-age children to care for them.

Mutual support between generations of one family is well documented. Parents provide childcare and household chores assistance to adult children who have become parents (Bucx, van Wel, & Knijn 2012; Yamato, 2017). Adult children also support their parents as they age, providing financial support, help with housework, care work and emotional support (Wu, 2021). Scholars have referred to this as “linked lives” to describe the reciprocal exchanges between adult children and their parents (e.g., Greenfield & Marks, 2006).

The intergenerational solidarity approach does not make gender-specific predictions, but empirical research reports gendered patterns in the intergenerational exchange. Analysing longitudinal data from the US, Silverstein et al. (2002) find that mothers on average provide more practical support including domestic help to adult children than fathers. Daughters also provide more support to parents than sons in the US (Silverstein et al., 2002) and the Netherlands (Kalmijin 2007). Similarly, in Canada daughters tend to provide more care than sons and the difference is particularly pronounced in traditionally female (and more time-consuming) tasks such as helping with personal care and domestic assistance (Campbell & Martin-Matthews, 2003). These patterns of intergenerational exchange in support indicate that gender is layered throughout the expressions of intergenerational solidarity. Consequently, in this paper, we propose to combine the intergenerational solidarity approach with the gender-centred perspective.

The *gender-centred* approach asserts that women perform more unpaid work and less of paid work compared to men because normative femininity and masculinity are associated with domestic work and paid work respectively (Berk, 1985; Brines, 1994; DeVault, 1990; Ferree, 1990; South & Spitze, 1994). By doing housework and caring for family members, women fulfil gendered social expectations and hence “do gender” (Brines, 19C94). Men in turn do not need to engage in unpaid work to establish their masculinity and in cases where their masculinity is damaged through loss of paid work, for example, they can withdraw from unpaid work to prevent further loss in masculinity (Brines, 1994). The gender-centred approach is rooted in marital-dyad assumptions and provides no indication as to how having two generations of adults in the household may influence the working-age couples’ time use (Geist & Ruppanner, 2018). However, if we extrapolate its assumptions from the marital dyad to a larger coresident household it suggests that older parents’ domestic help will not be necessarily a direct substitution of their children’s domestic work time because both older parents and adult children need to “do gender” in domestic work. An older mother or mother-in-law would assume some unpaid work responsibilities within the household as long as her health permits it to address her need to be feminine. For older adults, coresident fathers and fathers-in-law performing masculinity would mean avoiding unpaid work. Similarly, by the logic of *doing gender*, working-age men are not compelled to do unpaid work. Doing too much unpaid work could even damage their masculinity. Assuming that women are evaluated based on the result of their unpaid labour (e.g., clean home as found by Thébaud et al., 2019) rather than on the length of time they spend to achieve that result, multigenerational households have a clear potential for economies of scale through sharing the unpaid labour. According to the gender-centred approach, domestic work-sharing will largely happen between adult women and consequently the economies of scale will also mostly benefit women. Coresidence with older men is expected to increase adult women’s time spent on cooking, laundry, and cleaning associated with an additional adult in a household and these men are not expected to provide much help around the house themselves.

Before moving to discuss relevant empirical findings, we need to say a few words about the resource approach. Together with the gender-centred approach, this is a key theoretical approach widely used to make sense of paid and unpaid work participation within couples. It is well established that individual choices to allocate time to paid and unpaid work at least partially stem from resource-related factors, in which earning power and lack of time availability allow individuals to bargain away or outsource unpaid work to focus more on paid work (Gupta, 2007; Hook, 2017; Killewald & Gough, 2010). Economic approaches, however, do not appear to be equally useful for understanding dynamics in multigenerational households. While older parents and adult children are known to share resources, the older parents tend to be outside the labour market and hence do not have a similar incentive to focus on paid work and may not suffer from comparable time shortages. Consequently, while access to economic resources influences the ways working-age adults share paid and unpaid labour within couples, resource frameworks are less useful for understanding time allocation dynamics in multigenerational households. In this paper, we will control for resource availability, but we will not base our core hypotheses on the theories focusing on resources.

### Empirical Studies on Coresidence with Older Parents

In gender unequal societies, such as China, Japan, and Italy, women living in households with older parents are more likely to stay in paid work (Maurer-Fazio et al., 2011; Sasaki, 2002; Shen et al., 2016; Ta et al., 2018), but see (Yang et al., 2015; Yu & Xie, 2018) for contradicting evidence on China.

In another testimony to reductions in work-family conflict, living with husband’s parents is positively associated with the likelihood of having the first child in Taiwan (Chi & Hsin, 1996; Tsay & Chu, 2005), the second birth in South Korea (Yoon, 2017), and the first and second births in Japan (Fukukawa, 2013), but see some conflicting evidence for Japan and Italy (Raymo et al., 2010).

These studies are largely consistent with the intergenerational solidarity approach in which grandparents share unpaid work with their working-age children and children-in-law, making their domestic load more manageable and compatible with paid work. Existing research largely focuses on women, which is not surprising given that their domestic workload is heavier than men’s across the world (Gershuny & Kan, 2012; Kan & He, 2018; Kan et al., 2021; Sullivan et al., 2018). Women’s increased ability to maintain their attachment to the labour market and couples’ increased ability to have children suggest the flow of support goes from the older adults to the working-age generation. It is however possible that women’s greater ability to participate in the labour market and increased fertility are explained through reasons other than their sharing of domestic burden. If poorer households are more commonly choosing intergenerational living arrangements, then women’s greater likelihood of employment may stem from their greater need to work rather than from grandparental support (Raymo et al., 2010 find some support for this interpretation in Italy and Japan). Couples’ increased fertility in coresident households may be explained by the older adults’ greater power when it comes to family decision-making, rather than help they provide with unpaid work. There is little existing literature verifying whether living with older parents can reduce housework and childcare time and no explicit analysis making predictions about the way gender is (or isn’t) associated with the intergenerational exchange of unpaid labour. Exploring the dynamics in sharing domestic work by type of domestic work will lead to a better understanding of the associations between coresidence and paid work participation.

Finally, previous quantitative studies exclusively focused on women’s employment patterns and how these are associated with coresidence patterns, mentioning unpaid work only as a potential reason behind women’s paid work patterns, rather than analysing it directly. This assumes that the type of coresidence largely matters for women but not men and that intergenerational solidarity, when it happens, is only performed between different generations of women. Such a claim seems unlikely, and the role of both genders needs to be investigated.

In this paper, we will combine the gender-centred approach and the intergenerational solidarity theory and test the hypotheses listed below. The gender approach allows us to nuance the predictions of the intergenerational solidarity theory when it comes to such highly gendered behaviours such as participation in paid and unpaid labour.

Hypothesis 1: Coresidence with older mothers or mothers-in-law is associated with a reduction in unpaid work time and increase of paid work time of working-age women and, to a lesser extent, for men. The differences between working-age men and women will be especially pronounced in couples with children.

Hypothesis 2: Coresidence with older men is associated with longer unpaid work time and shorter paid work time for women.

Hypothesis 3: When older adults require care themselves (whether they are coresident or not), working-age women, but not men, will spend more time on unpaid domestic work and have less time on paid work.

### The Japanese Context

Coresidence of adult married children with older relatives has been falling in recent years in Japan, but it is still more common there than in European and Anglophone countries (Department of Economic & Social Affairs, 2017).

Recent research argues that this trend has at least partially been offset with the rise in proximate living (Kato, 2013; Wang & Raymo, 2020).

Japan is a particularly interesting context to investigate issues related to mutual support between generations. It is a highly developed and rapidly ageing country with a cultural environment where the traditional norm of filial piety – that also characterises other Asian countries – coexists with an emerging social norm of independence in old age (Takagi & Saito, 2013). On the policy level, in recent years Japan has shifted from de-familiasation policies that have been associated with a reduction in the family caring responsibilities to measures encouraging intergenerational coresidence (Izuhara, 2020).

At the same time, the rise of women’s educational attainment and employment rate has meant that working-age adults are exposed to greater work-family conflict. In 2018, Japan ranked 110^th^ out of 149 countries in the Global Gender Gap Report, a testament to its low levels of gender equality. Japanese wives continue to be responsible for virtually all housework and care work in married couples (Hertog et al., 2021) so the work-family conflict primarily affects them. Norms about gender roles have shown limited change among Japanese men and women born after the 1950ies (Piotrowski, Yoshida, Johnson, & Wolford, 2019). Behavioural change has also been slow. Average men’s and women’s paid work times and housework times changed only marginally between 1996 and 2016.

In Japan, motherhood is still most compatible with a traditional division of labour within families. Having children is associated with a dramatic rise in women’s unpaid work time, but not men’s (Sechiyama, 2013). Many women quit full-time employment when they have children and return to the labour market only several years later, often into dead-end jobs (Brinton & Oh, 2019).

Given the broad trends described above, it is not clear what coresidence implies for the working-age population in Japan in terms of participation in paid and unpaid labour. In this paper, we focus on the roles older adults play in their married children’s lives, unpacking the flows of intergenerational support from the perspective of adult children. As the arrival of children is associated with a rise in need for both unpaid work time and household income, we analyse families with and without children separately. Time use patterns of working-age adults in families without children offers a closer reflection of behavioural preferences. Paid and unpaid work time of working-age adults in families with children documents the extent to which the grandparents can make a difference in families with particularly high paid and unpaid workloads.

Our analysis is based on data from the 2006 Japanese Survey on Time Use and Leisure Activities, the latest Japanese national time use survey available abroad. The prevalence of coresidence between parents aged 65 and older and their adult children fell from 45% of all households with at least one member aged 65 or older to 39% of such households between 2005 and 2015. This limited change masks a much larger shift in the adult children’s circumstances. In 2005 21.3% of households with at least 1 member aged 65 or older were 3-generation households, i.e. households which contained the older adults, their married adult children and grandchildren. By 2015 this figure fell to 12.3%. This trend was partially offset by the rise in coresidence between the older adults and their adult children (both married and unmarried), who do not yet have children of their own (Cabinet Office, 2017).

As fewer married working-age adults choose multigenerational coresidence in Japan, it is possible, that the ones who do self-select into such an arrangement for a particular reason. Our data only contains information of non-coresident older parents when these parents require care and therefore, we cannot explore such parents’ contribution to their adult children’s families, while they are still healthy. As neighbourhood living is on the rise in Japan, this is an important limitation on the interpretability of our findings.

## Data and Methods

We analyse data from the 7^th^ wave of the Survey on Time Use and Leisure Activities (STULA) that was collected in mid-October 2006 (Statistics Bureau Japan, 2006).^1^ The survey is household-based and records information on all household members aged 10 or more. The survey collected information from 88,000 households or around 200,000 individuals from these households. Each respondent was requested to keep diary records of time use over two consecutive days. Survey data on time use is complemented with standard demographic and socio-economic indicators in the survey.

Our analytic sample consists of 23,226 heterosexual married couples where both spouses are aged 20 to 59 and are currently not in education. We excluded unmarried men and women as the core interest of this paper is to compare the difference multigenerational coresidence makes for working-age married men’s and women’s time use depending on whether they do or do not have children. In addition, single-parent households are considerably rarer in Japan than in most other develoVed countries and thus are a particularly selective group and their situation merits a separate analysis (OECD, 2016).

### Dependent Variables

We created continuous dependent variables for measuring the number of minutes spent on paid work, housework and childcare, and older-adult care. All these activities are based on the original activities as provided in the survey. Note that in STULA care for children over the age of 6 is categorised as “housework”. Therefore, in the present analysis, we regress household and childcare time together in the same set of models.

### Independent Variables

We estimate regressions separately for men and women with and without children under 20. Our core variable of interest is a combined measure of coresidence and care responsibilities, taking seven possible values: 1 “no care responsibilities and no coresident older parents” 2 “no care responsibilities, coresident with own or spouse’s mother” 3 “no care responsibilities, coresident with own or spouse’s father”, 4 “no care responsibilities, coresident both older parents or both older parents-in-law” 5 “nuclear household caring for non-coresident older adults” 6 “caring for a coresident older adults parent” 7 “other”.^2^ We use families with no coresident older adults as a reference point throughout the analysis. We do not separate households where working-age children co-reside with older adults for whom they care into separate types. We found few differences when we did this and the total sample households with coresident older adults requiring care is small so putting them all into one category increases the statistical power of the analysis.

We control for relevant covariates as follows: husband’s and wife’s ages (coded into 4 categories: “age 20–29”, “age 30–39”, “age 40–49” or “age 50–59”), household income (a continuous variable constructed using mid-points from 16 1-million yen bands available in the original survey), own and spouse's completed education (categorical variables with 3 levels: “high school or less”, “College or professional school” or “University”), a dummy for an urban household. Finally, we control for whether the day analysed is weekday or weekend.

We had no information on household income for 499 couples (< 2% of the total sample) and no full information on husband’s or wife’s education for 8 couples, there are no missing cases in our sample for other variables of interest. Household income is the only variable where missing observations exceeded 1% of our analytical sample. We imputed the missing values for household income by multiple imputations (i.e., from regression estimates obtained from other non-missing independent variables in our model). Model estimates from complete cases after listwise deletion were very similar (available upon request).

### Analytical Strategy

We start by presenting descriptive statistics of our dependent variables. We then follow Gimenez-Nadal and Molina’s approach (2013) and estimate linear seemingly unrelated regression (SUR) models (Zellner, 1962) on paid and unpaid work time by working-age husbands and wives. These models help us account for the fact that the time partners in a couple invest in paid and unpaid work is jointly determined. SUR models are more appropriate than Ordinary Least Squares (OLS) models because they allow us to conduct our analysis at the couple level rather than individual level. They also take account of the correlated error due to unobserved predictors in the equations concerning paid work time, housework time, and childcare time of both spouses. However, the SUR models do not allow us to take account of the repeated observations of individual diaries when calculating the standard error. We, therefore, keep one diary record for each respondent in our sample. Respondents completed time use diaries on 2 consecutive days from 14^th^ to 22^nd^ of October 2006. The specific date on which individuals started the survey was determined by the Statistics Bureau Japan staff based on the respondents' geographical location. We chose the first dairy record for all respondents. To check the robustness of our findings, we have conducted analyses on the second diary records of respondents. The results based on the first and the second diary records were not substantially different.

## Results

Looking at the paid and unpaid work patterns of married childless Japanese men and women (Table 3), wives do a lot more unpaid work compared to husbands, while husbands do a lot more paid work. Moreover, considerably more women compared to men report engaging in at least some unpaid work, while more men compared to women report engaging in paid work. To give an example: 98% of Japanese mothers report doing housework and childcare on a given day, compared to only 30% of fathers. On average mothers spend 370 min on housework and childcare daily, compared to 36 min contributed by fathers, a tenfold difference Tables 1, 2 and 3.

**Table 1:** Nuclear households and households including at least one older adults parent as a proportion of the total number of households in Japan between 2005 and 2015

	Total number of households 1,000 s	Nuclear families (%)	Families coresident with older parents (%)
2000	46,782	55.7	10.0
2005	49,063	57.7	8.9
2010	51,842	59.5	7.6
2015	53,332	60.6	6.2

Source: authors’ recalculation from Population Censuses data between 2000 and 2015 (Statistics Bureau Japan, various years)

**Table 2:** Married Couples Descriptive Statistics by Presence of Children

	No children under 20	Have children under 20		Full sample	
Coresidence pattern (%)					
* No care responsibilities*					
No coresident older adults	77.50		79.76		78.88
Coresident older mother	9.27		7.11		7.94
Coresident older father	1.68		1.50		1.57
Coresident older parents	5.38		9.08		7.64
* Caring for non-coresident older adults*	2.93		1.27		1.91
* Caring for coresident older adults*	2.42		0.89		1.48
* Other coresidence arrangements*	0.83		0.40		0.57
Wife’s Education (%)					
High School or less	63.70		52.61		56.90
College or Professional School	22.96		33.50		29.42
University	13.34		13.90		13.68
Husband’s Education (%)					
High School or less	61.04		52.47		55.78
College or Professional School	7.51		10.41		9.29
University	31.46		37.12		34.93
Lives in (%)					
Other	50.02		47.95		48.75
Lives in one of the 3 largest urban conglomerates	49.98		52.05		51.25
Day of the week (%)					
Weekday	71.02		72.13		71.70
Weekend	28.98		27.87		28.30
Wife's age (%)					
Age 20–29	6.88		10.55		9.13
Age 30–39	15.82		44.04		33.10
Age 40–49	19.39		38.45		31.06
Age 50–59	57.92		6.96		26.71
Husband's age (%)					
Age 20–29	4.77		6.41		5.78
Age 30–39	15.11		37.00		28.51
Age 40–49	13.84		39.76		29.71
Age 50–59	66.27		16.83		36.00
Household income (10,000 yen)	708		653		676
	(4.087)		(2.935)		(2.396)
Age of the youngest child (families with children under 20 only)			9		
			(0.056)		
*Weighted N of Couples*	9421		13,483		23,226

STULA 2006, percentages of individuals in each sub-category unless otherwise specified

**Table 3:** Married women's and men's paid and unpaid work in 2006 (limited people aged 20–64)

	Couples with no children under 20		Couples with children under 20	
	Minutes	Participation rate	Minutes	Participation rate
Wife: Paid Work	211.22	0.51	158.94	0.41
	(234.74)		(213.33)	
Housework and childcare	250.55	0.94	369.73	0.98
	(169.95)		(201.91)	
Older-adult care	5.30	0.04	4.12	0.04
	(36.31)		(31.71)	
Husband: Paid Work	440.41	0.79	478.18	0.81
	(264.75)		(272.49)	
Housework and childcare	23.48	0.23	36.49	0.30
	(64.82)		(87.36)	
Older-adult care	1.28	0.01	0.79	0.01
	(18.00)		(11.79)	
*Weighted N*	9436	9436	13,821	13,821

Source: Survey of Time Use and Leisure Activities 2006

**Fig. 2:**
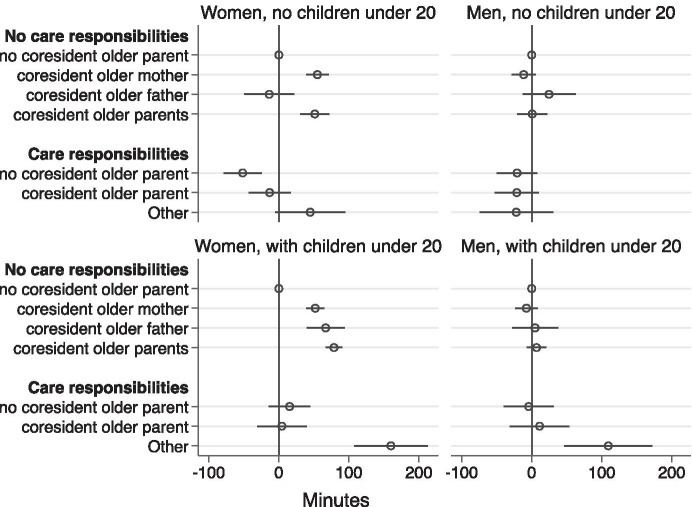
Married women's and men's paid work time coresidence pattern coefficients

**Fig. 3:**
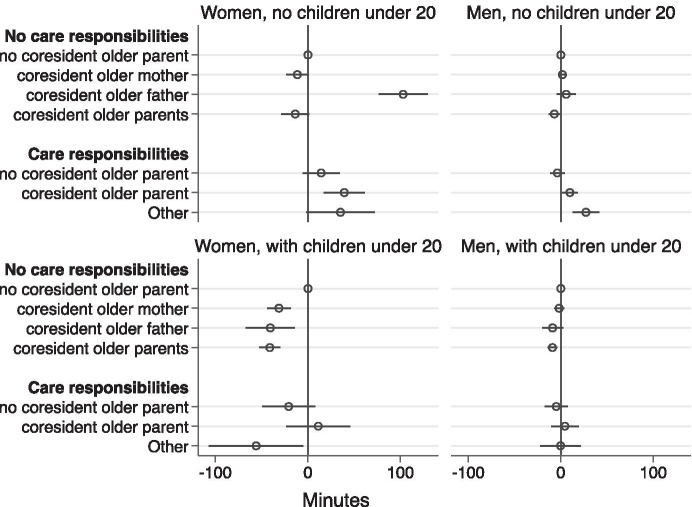
Married women's and men's housework and childcare time coresidence pattern coefficients

**Fig. 4:**
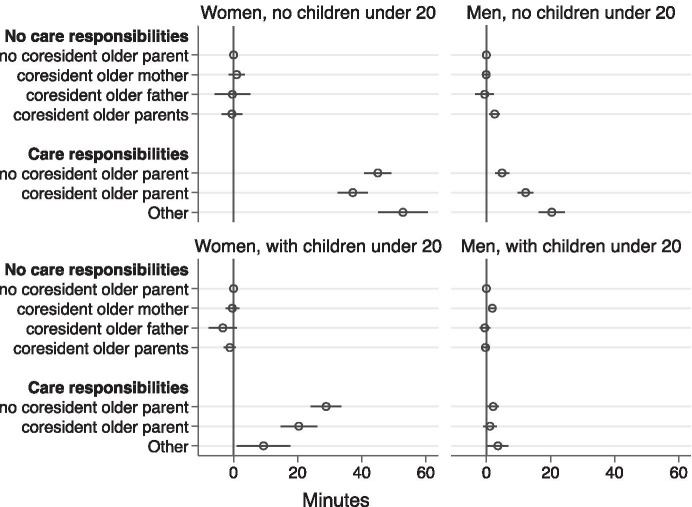
Married women's and men's older-adult care time coresidence pattern coefficients

We present the results of SUR models in Tables 4 and 5. Figures 2, 3, and 4 summarise the coefficients and standard error of our coresidence and care measure in these models. In the first set of models, we compare husband’s and wives’ paid work time in couples with and without children (see Fig. 2 and Tables 4 and 5).

**Table 4:** Married childless women's and men's time in minutes in 2006 (limited to individuals aged 20–59)

	Wives			Husbands		
	Work	Housework + childcare	Older-adult care	Work	Housework + childcare	Older-adult care
*No care responsibilities* No coresident older adults	–	–	–	–	–	–
Coresident older mother	55.12*** (8.13)	− 11.49 (6.05)	0.97 (1.26)	− 11.53 (8.61)	1.77 (2.30)	− 0.08 (0.66)
Coresident older father	− 13.74 (18.20)	102.88*** (13.53)	− 0.35 (2.83)	24.67 (19.28)	5.76 (5.16)	− 0.57 (1.47)
Coresident older parents	51.36*** (10.42)	− 13.73 (7.75)	− 0.50 (1.62)	0.67 (11.03)	− 7.01* (2.95)	2.57** (0.84)
*Caring for non − coresident older adults*	− 51.63*** (13.85)	14.31 (10.29)	45.02*** (2.15)	− 20.92 (14.67)	− 3.77 (3.92)	4.87*** (1.12)
*Caring for coresident older adults*	− 13.07 (15.21)	39.28*** (11.31)	37.22*** (2.36)	− 21.29 (16.11)	9.80* (4.31)	12.23*** (1.23)
*Other coresidence arrangements*	44.84 (25.31)	35.17 (18.82)	52.86*** (3.93)	− 22.08 (26.81)	27.15*** (7.17)	20.40*** (2.04)
*Wife’s age* Age 20–29	–	–	–	–	–	–
Age 30–39	− 22.48 (12.80)	17.17 (9.51)	1.56 (1.99)	− 40.64** (13.55)	− 1.20 (3.62)	0.05 (1.03)
Age 40–49	35.35* (16.02)	− 11.78 (11.91)	0.81 (2.49)	− 47.82** (16.97)	− 0.05 (4.54)	0.63 (1.29)
Age 50–59	30.85 (17.09)	3.52 (12.71)	3.18 (2.66)	− 64.33*** (18.11)	1.56 (4.84)	0.26 (1.38)
*Husband’s age* Age 20–29	–	–	–	–	–	–
Age 30–39	42.05** (14.37)	− 15.35 (10.69)	− 0.10 (2.23)	14.85 (15.22)	2.40 (4.07)	0.19 (1.16)
Age 40–49	− 8.42 (17.23)	41.01** (12.81)	0.79 (2.68)	41.81* (18.25)	− 7.20 (4.88)	0.08 (1.39)
Age 50–59	− 12.35 (18.47)	54.42*** (13.73)	1.81 (2.87)	− 11.24 (19.56)	− 2.01 (5.23)	1.26 (1.49)
*Wife’s education* High School or less	−	−	−	−	−	−
College or Professional School	13.78* (6.15)	− 8.13 (4.57)	0.28 (0.96)	− 7.14 (6.51)	1.26 (1.74)	1.30** (0.50)
University	67.82*** (8.21)	− 44.00*** (6.10)	2.34 (1.28)	− 36.21*** (8.69)	3.64 (2.32)	1.26 (0.66)
*Husband’s education* High School or less	−	−	−	−	−	−
College or Professional School	− 8.33 (9.30)	9.87 (6.92)	− 1.84 (1.45)	5.31 (9.85)	2.33 (2.63)	− 0.68 (0.75)
University	− 32.45*** (6.07)	9.01* (4.51)	− 1.25 (0.94)	− 4.28 (6.43)	1.26 (1.72)	− 0.44 (0.49)
Logged household income	13.63*** (3.75)	− 2.44 (2.79)	− 2.29*** (0.58)	25.57*** (3.97)	− 3.20** (1.06)	− 0.12 (0.30)
Weekday	–	–	–	–	–	–
Weekend	− 122.28*** (5.11)	5.82 (3.80)	1.83* (0.79)	− 254.43*** (5.41)	32.76*** (1.45)	− 0.11 (0.41)
Lives in one of 3 large urban conglomerates	− 63.70*** (4.83)	44.25*** (3.59)	2.06** (0.75)	− 15.97** (5.11)	1.75 (1.37)	− 0.48 (0.39)
Constant	164.87*** (24.42)	203.50*** (18.16)	12.24** (3.80)	420.57*** (25.87)	33.24*** (6.92)	0.17 (1.97)
Observations	9260					
*R*^2^	0.0976	0.0512	0.0909	0.2063	0.0592	0.0262

Standard errors in parentheses Source: Survey of Time Use and Leisure Activities 2006 *p < 0.05, **p < 0.01, ***p < 0.001

**Table 5:** Married mothers' and fathers' time in minutes in 2006 (limited to individuals aged 20 − 59)

	Wives			Husbands		
	Work	Housework + childcare	Older − adult care	Work	Housework + childcare	Older − adult care
*No care responsibilities* No coresident older adults	–	–	–	–	–	–
Coresident older mother	48.34*** (6.72)	− 27.51*** (6.48)	− 0.31 (1.10)	− 9.79 (8.18)	− 0.13 (2.83)	1.71*** (0.40)
Coresident older father	57.86*** (14.33)	− 34.62* (13.82)	− 3.81 (2.35)	− 0.46 (17.43)	− 6.60 (6.03)	− 0.47 (0.85)
Coresident older parents	79.26*** (6.25)	− 40.94*** (6.03)	− 1.15 (1.03)	8.77 (7.60)	− 8.90*** (2.63)	− 0.24 (0.37)
*Caring for non-coresident older adults*	10.12 (14.85)	− 14.14 (14.32)	28.81*** (2.44)	− 5.07 (18.06)	− 3.35 (6.25)	2.10* (0.88)
*Caring for coresident older adults*	3.08 (18.33)	13.23 (17.68)	21.17*** (3.01)	8.41 (22.30)	5.76 (7.71)	1.20 (1.08)
*Other coresidence arrangements*	162.06*** (27.37)	− 68.59** (26.40)	7.24 (4.49)	101.57** (33.30)	− 2.08 (11.51)	3.90* (1.61)
*Wife’s age* Age 20–29	−	−	−	−	−	−
Age 30–39	− 17.24* (7.49)	27.03*** (7.22)	0.34 (1.23)	0.44 (9.11)	− 11.68*** (3.15)	− 2.56*** (0.44)
Age 40–49	− 31.51*** (9.44)	47.20*** (9.10)	− 1.58 (1.55)	− 9.09 (11.48)	− 7.62 (3.97)	− 2.53*** (0.56)
Age 50–59	− 25.00* (12.19)	44.52*** (11.75)	− 0.34 (2.00)	− 60.62*** (14.82)	− 2.98 (5.13)	− 3.08*** (0.72)
*Husband’s age* Age 20–29	−	−	−	−	−	−
Age 30–39	19.08* (9.07)	− 17.85* (8.74)	0.37 (1.49)	3.69 (11.03)	12.91*** (3.81)	2.98*** (0.53)
Age 40–49	34.02** (10.35)	− 25.43* (9.98)	2.79 (1.70)	− 2.98 (12.59)	7.48 (4.35)	2.67*** (0.61)
Age 50–59	31.65** (11.70)	− 24.67* (11.28)	2.33 (1.92)	− 21.18 (14.23)	10.35* (4.92)	2.97*** (0.69)
*Wife’s education* High School or less	–	–	–	–	–	–
College or Professional School	1.17 (3.96)	4.01 (3.82)	0.54 (0.65)	− 3.77 (4.82)	1.14 (1.67)	0.84*** (0.23)
University	10.44 (5.75)	14.31** (5.54)	1.22 (0.94)	− 26.97*** (6.99)	22.17*** (2.42)	1.32*** (0.34)
*Husband’s education* High School or less	–	–	–	–	–	–
College or Professional School	− 17.73** (5.84)	29.52*** (5.64)	− 0.21 (0.96)	− 3.44 (7.11)	5.61* (2.46)	− 0.75* (0.34)
University	− 26.29*** (4.21)	32.06*** (4.06)	− 0.22 (0.69)	− 4.74 (5.13)	8.99*** (1.77)	− 0.25 (0.25)
Logged household income	12.38*** (3.26)	− 11.05*** (3.15)	− 0.60 (0.54)	11.79** (3.97)	− 2.42 (1.37)	− 0.56** (0.19)
Weekday	–	–	–	–	–	–
Weekend	− 117.48*** (3.75)	− 11.85** (3.61)	− 1.54* (0.62)	− 301.19*** (4.56)	52.70*** (1.58)	0.42 (0.22)
Youngest child's age	8.17*** (0.43)	− 10.32*** (0.42)	0.11 (0.07)	1.61** (0.53)	− 2.40*** (0.18)	0.01 (0.03)
Lives in 3 large urban conglomerates	− 40.76*** (3.45)	25.97*** (3.33)	− 0.61 (0.57)	-7.34 (4.20)	0.61 (1.45)	0.04 (0.20)
Constant	51.72** (20.07)	501.19*** (19.36)	5.93 (3.30)	493.50*** (24.42)	51.77*** (8.44)	3.30** (1.18)
Observations	13,336					
*R*^2^	0.1721	0.1315	0.0172	0.2534	0.1289	0.0082

Standard errors in parentheses Source: Survey of Time Use and Leisure Activities 2006 *p < 0.05, **p < 0.01, ***p < 0.001

## Paid Work Time

As can be seen in Table 4 and Fig. 2, in most families coresidence with healthy older parents or parents-in-law is associated with longer time spent at work for wives. However, for wives without underage children, coresidence with an older father makes no significant difference to paid work time compared with wives who do not live with older parents. Compared to wives in families with no children under 20 who do not live with older parents, wives who co-reside with both older parents (or parents-in-law) or with an older mother (or mother-in-law) alone have 51 min and 55 min longer paid work time per day respectively. Referring to Table 5 and Fig. 2, in the case of mothers, all types of coresidence with healthy older parents: both parents, older mothers only, and older fathers only, increase their daily work time by 79 min, 48 min, 58 min per day respectively compared with mothers who do not live with older parents. When older parents require care themselves, coresidence stops being a career boost for women. Referring again to Table 4, for women without underage children and with caring responsibilities for non-resident older parents, their paid work time is 52 min per day shorter compared with women with no underage children, no coresident older adults and no care responsibilities. From Tables 4 and 5, interestingly, caring for coresident older adults makes no significant difference to paid work time for both childless women and mothers compared with women who have no care responsibilities.

Referring to Tables 4 and 5 and Fig. 2, we can see that in all types of families, coresidence with older parents does not make any significant difference to the paid work time of childless husbands and fathers compared with their counterparts who do not live with older parents.

## Housework and Childcare Time

Figure 3 and Tables 4 and 5 show the associations between housework and childcare time and types of coresidence with older parents. For women with no children under 20, there is no significant difference to housework and childcare time among those who co-reside with healthy older mothers and mothers-in-law alone, those who co-reside with older parents or parents-in-law, and those who do not live with older parents. Remarkedly, childless women residing with their fathers or fathers-in-law alone spend 103 min more on housework and childcare per day compared with childless women who do not live with older parents.

As for men who have no children under 20, coresidence with older parents makes little or no change to their housework and childcare time compared with men who do not live with older parents (except for childless men who live with older parents, whose housework and childcare time is 7 min less per day).

Furthermore, for both childless men and women, there is no significant difference in housework and childcare time between those who have caring responsibilities for non-resident older adults and those who do not live with older parents and have no caring responsibilities. However, caring responsibilities for coresident older parents will increase women’s and men’s housework and childcare time by 39 min and 10 min per day respectively, compared with their counterparts who do not live with older parents and do not have caring responsibilities.

Turning to the results on parents, coresidence arrangements make significant differences to the housework and childcare time of mothers. Referring to Table 5 and Fig. 3, we can see that coresidence with older parents, older mothers or mothers-in-law only and older fathers or fathers-in-law only is associated with 41 min, 28 min and 35 min less in housework and childcare time per day respectively compared with mothers who do not live with older adults.

As for fathers, similar to the results of childless men, coresidence with older adults arrangements make little or no difference to their daily housework and childcare time compared with fathers who do not live with older parents (except for fathers who live with their older fathers or fathers-in-law only, whose daily housework and childcare time is 9 min less).

Furthermore, for mothers and fathers, caring responsibilities for resident or non-resident older adults make no significant difference to the housework and childcare time compared to their counterparts who do not live with older parents and do not have caring responsibilities.

## Older-Adult Care Time

Finally, Fig. 4 and Tables 4 and 5 show the patterns of older-adult care time reported by all married men and women. For women, it is the care responsibilities rather than the coresidence arrangements which make a difference to their older-adult care time. Coresidence with older parents, mothers or mothers-in-law only, or fathers or fathers-in-law only is not significantly associated with older-adult care time for women, regardless of their parental status, compared with their counterparts who do not live with older parents and have no older-adult care responsibilities. However, childless women who care for non-coresident older adults and those who care for coresident adults spend 45 and 37 min per day more respectively on older-adult care compared with childless women who do not live with older parents and have no older-adult care responsibilities. The figures for childless men are respectively 5 min and 12 min per day more. Childless men who co-reside with healthy older parents also spend 3 min longer on older-adult care per day compared with childless men who do not live with their older parents and have no older-adult care responsibilities.

As for parents, the older-adult care time difference between those who have older-adult care responsibilities and those who do not are less than childless men and women. For mothers, those who care for non-coresident older parents and those who care for coresident parents spend 29 min and 21 min per day more on older-adult care than those who do not live with older parents and have no older-adult care responsibilities. As for fathers, older-adult care responsibilities are associated with 2 min longer per day for those who have care responsibilities for non-resident parents compared with those who do not live with their older parents and have no caring responsibilities.

Nonetheless, we should note that women’s older-adult care time varies more substantially according to the older-adult care responsibilities than men’s.

Overall, the results broadly support Hypothesis 1: coresidence with older parents or older mothers or mothers-in-law alone is largely associated with increases in women’s paid work time and decreases in mothers’ housework and childcare time. Furthermore, coresidence with older parents or older mothers alone makes little or no difference to men’s paid work time and housework and childcare time.

We find contradictory evidence for Hypothesis 2. Coresiding with a single healthy older father or father-in-law is associated with longer, rather than shorter, paid work time for mothers. It is also associated with shorter, rather than longer, housework and childcare time for mothers but significantly longer housework and childcare time for childless women. In this paper, we have a combined measurement of housework and childcare time. Therefore, these findings may reflect the fact that some older men are less willing or able to help with housework. However, they might compensate with childcare help in families with children. It might also be the case that as previous studies suggest, men find childcare more interesting and enjoyable than housework (Gershuny, 2012; Gershuny & Sullivan, 2019; Poortman & Van Der Lippe, 2009). So in the case of women with underage children, the older fathers or fathers-in-law may prefer to spend time on childcare rather than housework when they offer their domestic support to their children. In sum, we find clear gendered patterns between working-age men and women in terms of the difference coresidence makes for their daily routines.

When it comes to the gendered patterns of support provided by the older adults, the results show that older mothers and older mothers-in-law provide much more consistent support than older fathers and older fathers-in-law in alleviating married women’s work-life balance: by increasing the mothers’ and childless women’s paid work time and reducing mothers’ housework and childcare time. These findings are broadly consistent with the intergenerational solidarity theory and the gender-centred perspective.

We also find mixed results for Hypothesis 3. Childless women’s paid work time will be reduced when they care for non-resident older parents and their housework and childcare time will be increased when they care for coresident older parents. However, we find insignificant associations in the case of both mothers and fathers. Moreover, childless men’s housework and childcare time will be increased slightly if they care for coresident older parents.

Nevertheless, concurring with Hypothesis 3, we have found strong gendered patterns of associations between older-adult care responsibilities and older-adult care time. Although both married men and women spend more time on older-adult care if they report older-adult care responsibilities, the increase in older-adult care time is much larger for women.

## Conclusion

This paper has provided a comprehensive account of variation in married men’s and women’s paid work, housework and childcare, and older-adult care time using large scale national survey data of Japan. It contributes to the literature of the domestic division of labour by emphasising the roles of older parents. Previous studies focussed predominantly on the dyadic interactions between husbands and wives. Yet in East Asia it is common for young married couples to co-reside with their older parents or parents-in-law.

We have found strong gendered patterns of associations between older parents’ coresidence arrangements and paid work, housework and childcare, and older-adult care time of working-age couples. The findings suggest that gendered expectations and gendered norms play an important role in the division of labour and inter-generational support in multigenerational households in Japan. Unpaid domestic work is still largely women’s work. Coresidence with older parents helps working-age women more than men, to alleviate work-family conflicts. Furthermore, older mothers provide married couples with more consistent and substantial support in childcare and housework than older fathers do.

We are also the first to integrate intergenerational solidarity and gender perspectives to examine the phenomena and demonstrate that gender roles play an important role when it comes to intergenerational support. Specifically, we evaluated how working-age married men and women benefit from coresidence with the older adults and how they might adjust their daily lives when the older adults need care themselves.

We paid particular attention to variation by the presence of children. Families without children are less subject to income and time crunch pressures that encourage Japanese mothers and fathers to conform to traditional family norms. Consequently, time use patterns of husbands and wives in families without children are a better indicator of behavioural preferences while time use patterns in families with children are a better indication of how much of a difference older adult relatives can make to their children’s work-life balance.

Our findings are consistent with intergenerational solidarity theory, but also highlight its insufficiency in explaining the flows and the types of support between the generations. First, as intergenerational solidarity theory would predict, living with older relatives is associated with working-age men’s and women’s ability to spend more time working and less time on unpaid work. This suggests that the older adults provide support with domestic responsibilities and let their adult children keep a stronger attachment to the labour market. However, the findings also suggest that men and women co-residing with older relatives increase their paid work time by more than they decrease their unpaid work time, indicating that the older adults promote labour market attachment not only through direct help with housework but also (potentially) by covering time-sensitive tasks like picking up children from school or accompanying children to afterschool activities. This would enable working-age adults to work longer hours and catch up with the rest of domestic work at a time convenient for them.

We see that married women’s paid and unpaid work participation is much more sensitive to coresidence arrangements and to care responsibilities compared to their husbands, as predicted by the gender centred approach. Japanese women spend longer time on unpaid work compared to men and have a weaker attachment to the labour market; the patterns of intergenerational exchange are based on the traditional gendered division of labour. Coresidence with older adults is an important way to alleviate work-family conflict for working-age women, while for men in most cases it only makes a difference of a couple of minutes a day.

Improving our knowledge of how coresidence influences work-life balance for working-age married men and women is essential at the time when coresidence trends are experiencing dramatic changes throughout the world. In Japan, coresidence rates are going down and 3-generation households are disappearing at a particularly fast rate (Cabinet Office, 2017). Our findings highlight the need for a better understanding of the implications of the increasing number of working-age married men and women who will not co-reside with their older relatives. Further studies should investigate whether these couples seek support from older parents living near their homes instead. Coresidence is associated with a large increase in time married women spend in paid work, as fewer women co-reside with their parents and in-laws there is a need for policies to replace older adults’ support their daughters’ and daughters’-in-law labour market attachment.

One limitation of our analysis is the shortcomings stemming from the limited information about the background characteristics of the respondents and the cross-sectional nature of the data. We have no information about the past behaviours of our respondents, their health status and characteristics of their non-coresident family members. It is possible, that some of the coresident adult children choose to move in with parents to achieve the time use patterns observed, and it is the background characteristics of such individuals (e.g., career drive), that offer a better explanation for the observed time use patterns (e.g., longer work hours) than the type of coresidence. The cross-sectional data only allow us to test the associations, rather than casual links, between residential arrangements and the gender division of labour of the working-age couples. Couples may be self-selected into living with older parents for a particular reason. Our data only contains information of non-coresident older parents when these parents require care and therefore, we cannot explore such parents’ contribution to their adult children’s families, while they are still healthy. As neighbourhood living is on the rise in Japan, this is an important limitation on the interpretability of our findings. Future research should employ longitudinal data to investigate how coresidence arrangement might change across life course stages and how this is related to the division of paid work and unpaid work between spouses.

In this paper, we demonstrate how the intergenerational solidarity theory and the gender centred approach can complement each other in explaining time use patterns across generations. Japan, however, is characterised by particularly high gender inequality in households. Future research should investigate if the two theories are similarly complementary in countries characterised by greater gender equality.

Notably, here we focussed on the perspective of working-age adults. Of course, what benefits working-age adults may not necessarily be beneficial for their older relatives. As highlighted by Carr and Utz (2020) there are generational asymmetries in perceptions and reporting of mutual experiences. Future research should analyse coresidence from the perspective of older men and women. Such research will clarify the relevance of intergenerational solidarity theory and gender-centred approach for time use on the side of the older generation in co-residing households. It will also highlight potential benefits and burdens of coresidence for older adults as more and more of them choose to live independently in Japan.

## Footnotes

1 The latest 9^th^ wave of the Survey on Time Use and Leisure Activities (STULA) that was collected in mid-October 2016 is only available in Japan. We use the 7^th^ wave, as this is the latest wave available to researchers outside Japan.

2 The “other” category includes individuals who (1) report caring for an over 65-year-old person, who is not one’s parent (e.g., one’s spouse) and (2) individuals who coreside with healthy older adults and provide care for non-coresident older adults (3) individuals with multiple care locations (caring for a coresident and non-coresident older adults).
